# Electrochemical Disinfection of Dental Implants – a Proof of Concept

**DOI:** 10.1371/journal.pone.0016157

**Published:** 2011-01-14

**Authors:** Dirk Mohn, Matthias Zehnder, Wendelin J. Stark, Thomas Imfeld

**Affiliations:** 1 Institute for Chemical and Bioengineering, Department of Chemistry and Applied Biosciences, ETH Zurich, Zurich, Switzerland; 2 Department of Preventive Dentistry, Periodontology, and Cariology, University of Zurich Center of Dental Medicine, Zurich, Switzerland; Institut de Pharmacologie et de Biologie Structurale, France

## Abstract

**Background:**

Peri-implantitis has gained significant clinical attention in recent years. This disease is an inflammatory reaction to microorganisms around dental implants. Due to the limited accessibility, non-invasive antimicrobial strategies are of high interest. An unexpected approach to implant disinfection may evolve from electrolysis. Given the electrical conductivity of titanium implants, alkalinity or active oxidants can be generated in body fluids. We investigated the use of dental titanium implants as electrodes for the local generation of disinfectants. Our hypothesis was that electrolysis can reduce viable counts of adhering bacteria, and that this reduction should be greater if active oxidative species are generated.

**Methodology/Principal Findings:**

As model systems, dental implants, covered with a mono-species biofilm of *Escherichia coli* C43, were placed in photographic gelatin prepared with physiological saline. Implants were treated by a continuous current of 0 - 10 mA for 15 minutes. The reduction of viable counts was investigated on cathodes and anodes. In separate experiments, the local change in pH was visualized using color indicators embedded in the gelatin. Oxidative species were qualitatively detected by potassium iodide-starch paper. The *in situ* generated alkaline environment around cathodic implants caused a reduction of up to 2 orders of magnitude in viable *E. coli* counts. On anodic implants, in contrast to cathodic counterparts, oxidative species were detected. Here, a current of merely 7.5 mA caused complete kill of the bacteria.

**Conclusions/Significance:**

This laboratory study shows that electrochemical treatment may provide access to a new way to decontaminate dental implants *in situ*.

## Introduction

Peri-implantitis is an inflammatory process of the tissues around an osseointegrated oral implant in function and results in loss of the supporting bone [1]. Bacterial colonization of dental implants and the infection of peri-implant tissues can lead to chronic bone destruction and may consequently lead to implant failure [2]. Current methods to treat peri-implantitis include mechanical decontamination and local antiseptic or antibiotic treatment (for reviews, see [3] and [4]). Suggested implant surface treatments are scaling, CO_2_-lasers, air abrasive powder, chlorhexidine or hydrogen peroxide irrigation, or local antibiotics. At present, most of the information on the effectiveness of such interventions derives from case reports, so that no evidence-based consensus has been reached as to which option is clinically most advantageous.

**Figure 1:**
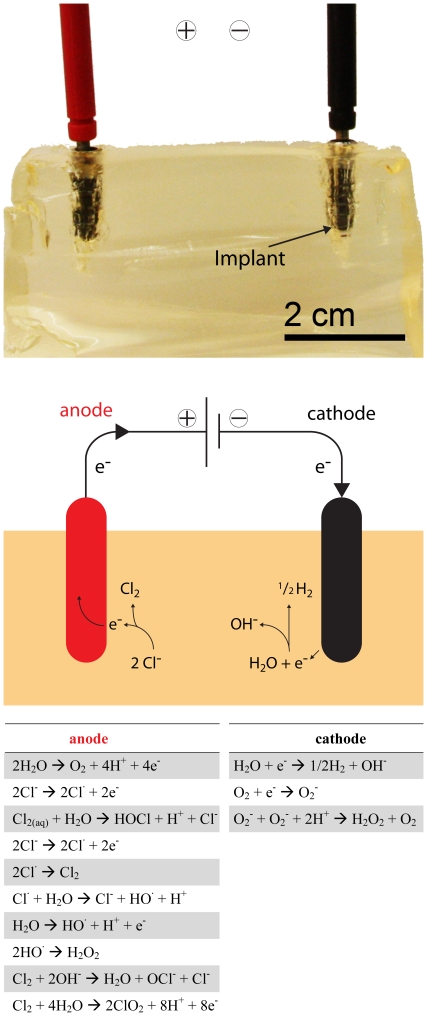
Simulated soft tissue (gelatin) with two dental implants. The anode (top, left) and cathode (top, right) were connected as part of an electric circuit powered by an external controller (top). The most probable reactions occurring at the electrodes are displayed in a scheme of the electrolysis setup to further illustrate the process (middle) and an overview (below) of the most likely occurring reactions is shown, too (adapted from [8], [19]).

In spite of the finding that the pattern of spread of inflammation is different in periodontal and in peri-implant tissues [5], most of the proposed debridement protocols for dental implants have been derived from periodontology. There is, however, a pronounced difference between dental implants and teeth, namely the fact that the former are made of titanium, an electrically conducting metal. Accordingly, a conceivable alternative minimally invasive approach to reduce the number of viable microorganisms on dental implants could be electrochemistry. This purification process is well-known for water disinfection [6]–[9] and uses the possibility to generate active substances on-site on the electrode. Neat water can be decomposed into oxygen and hydrogen. At the cathode, water is decomposed into hydrogen and hydroxide ions, which creates an alkaline environment (high pH). At the anode, oxygen and protons are generated (low pH). In the presence of chloride ions (i.e. in a physiological environment) additional highly active oxidizing agents such as chlorine (Cl_2_), HOCl, OCl^−^ or ClO_2_ are generated (Fig. 1). These are the key species involved in electrochemical disinfection [8], [10]. The formation of oxidative substances further depends on the quality and material of the electrode [11] and current/voltage. It has been reported for catheter disinfection that low amperage electric current can effectively inhibit bacterial growth [12]. The so-called iontophoresis makes use of microampere currents and has been applied successfully in an urinary catheter system [13], [14]. In both applications, the electrodes are in an undivided electrochemical cell, whereas in this work, two different and spatially separated environments are generated for the reduction of adhered microorganisms. In order to model a situation in a gingiva/implant site (patient), we used a physiological gelatin block with physiological saline and titanium implants produced for oral application.

This study therefore describes the electrochemical reduction of adhered bacteria on dental implants. Also, the hypothesis that viable count reduction would be greater at the anode was tested. We expected oxidative substances generated at the anode to have a higher disinfecting capacity than the mere alkaline environment around cathodes.

## Materials and Methods

### Implant disinfection

To examine the possibility of using electric current to eliminate or at least significantly reduce bacteria on implant surfaces, standard dental titanium implants (Straumann SLA, Straumann AG, Basel, Switzerland) were coated with a mono-species biofilm of *Escherichia coli* (strain C43). Enteric bacteria such as *E. coli* are amongst the species most frequently isolated from peri-implantitis sites [15]. A mono-species biofilm was created according to a published protocol [16]. In brief, implants of 4.1 mm in diameter and 12 mm in length were autoclaved prior to application. A total of 9 implants were used for this study. Specimens were immersed in 1.7 ml horse serum (BioWhittaker, Walkersville MD, USA) diluted (1/10) in physiological saline (0.9% NaCl) for 2 hrs at 37°C in Eppendorf tubes to create a protein film on their surface. *E. coli* was grown in Difco LB Broth (Chemie Brunschwig, Basel, Switzerland), and diluted to 7.0–7.5 log_10_ colony-forming units (CFU)/ml prior to immersion in saline. Implants were immersed two times for 60 hrs in 1.7 ml of the *E. coli* suspension at 37°C. After 60 hrs each set of implants was dipped thrice in MilliQ water to remove the loosely adherent bacteria. To imitate inflammatory soft tissues around the implant, photographic gelatin (Ballistic grade A, Gelita, Eberbach, Germany) was prepared according to the manufacturer's guideline with saline (20 wt% gelatin and 80 wt% saline). Holes were cut and filled with 20–40 µl of saline to ensure good contact of the implant with gelatin. Implants were placed in gelatin blocks with a space of 4 cm in between, and the electric circuit was connected (Fig. 1). Implants without electric circuit served as positive controls (maximal recovery of bacteria). Electrochemical treatments were done in triplicates. On each experimental day, implants were assigned to new treatment groups. A continuous current of 2 mA, 5 mA, 7.5 mA or 10 mA was applied for 15 min. Subsequently, the implants were immersed in 1.7 ml of saline, vortexed for 30 sec and ultrasonicated for 5 sec (80 W, UP400S, Hielscher, Teltow, Germany) to remove adhering microorganisms. Dilution series were plated on tryptic soy agar plates (VWR, Sentmenat, Barcelona, Spain) and incubated for 12 hrs at 37°C, revealing the reduction of viable bacteria compared to positive control treatments (no current). Purity of growth was checked by assessing colony morphology and by Gram staining.

Data pertaining to viable *E. coli* counts are presented as log_10_ colony-forming units (CFU). Mean values between test and control treatments at each time point were compared using one-way analysis of variance (ANOVA) followed by Bonferroni's correction for multiple testing. The alpha-type error was set at 0.05.

### Visualization of electrolysis

The gelatin around the implant was analyzed using pH indicators and a calibrated pH electrode (Seven Easy, Mettler-Toledo, Greifensee, Switzerland). Gelatin was produced as described above, using two indicators (thymol blue and bromocresol green) to visualize the pH change in the environment around the implant. Implants were treated with 10 mA for 15 minutes. Then, bigger holes (9 mm) were cut in the ballistic gelatin blocks (no indicator) to use larger amounts of saline in order to be able to check the pH precisely using a calibrated electrode. To this end, a treatment with 10 mA and 15 min was carried out while the pH was measured every minute. During measurement of the pH the current was turned off and the implants were removed so as to not influence the pH electrode.

As suggested in the literature, oxidative species are generated at the anode. Potassium iodide-starch paper, that shows the color dark blue if exposed to oxidants like chlorine, was placed above both electrodes to check the formation of oxidative species. Eppendorf tubes were used to cover the paper and the implant and to capture evolved oxidants.

## Results

**Figure 2:**
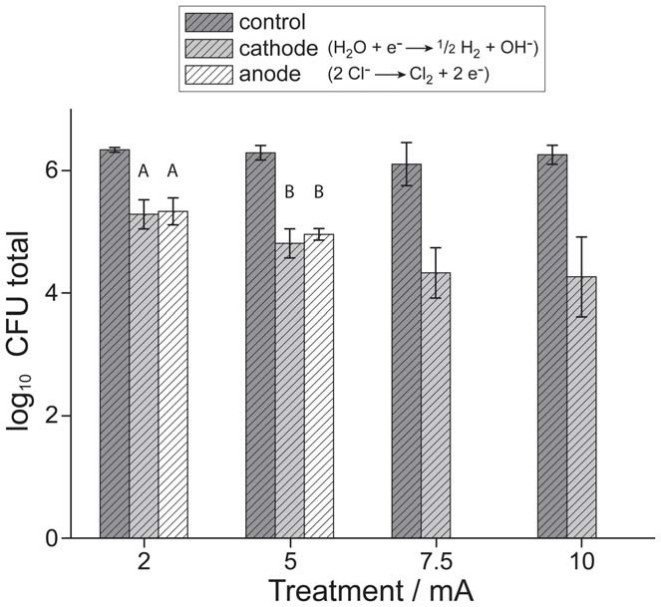
Reduction of *E. Coli* adhered on dental implants. Viable *E. coli* counts were reduced on implants after current treatment for 15 minutes (mean log_10_ CFU (colony-forming units) values (N = 3) and standard deviation). Pronounced differences arised for anodic (oxidative environment) and cathodic (alkaline environment) implants. A full disinfection could be obtained if an implant was used as anode and at a current of at least 7.5 mA. Same capital letters above data sets indicate no statistically significance (p>0.05).

Standard dental implants served as electrodes for the electrolysis of physiological saline around the dental implants and in the simulated soft tissue (gelatin). In contrast to the positive control treatment, i.e. when no current applied, both anode and cathode electrodes induced a reduction of bacterial counts for different currents during the 15 minutes treatment (Fig. 2). At the cathode, viable bacteria decreased with increasing current. At the maximum current of 10 mA, viable bacteria were reduced by 2 orders of magnitude (99% in total counts) compared to the positive control. The difference in log_10_ CFU was significant (p<0.05) compared to the control treatment already with a continuous current of 2 mA. The electrochemical disinfection at the anode caused a statistically similar reduction of *E. coli* counts at 2 and 5 mA. A treatment with 7.5 mA or 10 mA resulted in a complete kill of all viable bacteria and complete disinfection (Fig. 2, p<0.05 compared to log_10_ CFU values at the cathode and implants with no current applied).

**Figure 3:**
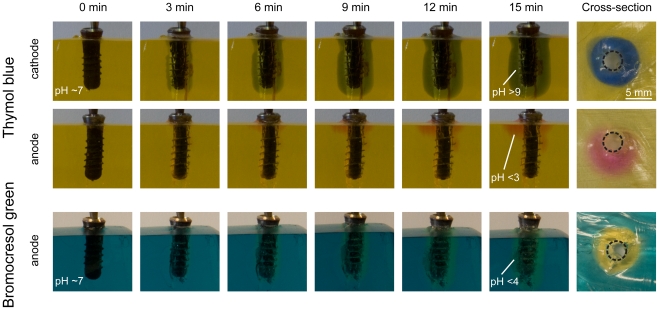
Proof of pH changes during electrochemical implant treatment. Photographic images of dental implants in simulated soft tissue using pH-sensitive dyes to visualize local pH changes. The dark blue color for thymol blue indicated a pH above 9 (alkaline) while the pink color confirmed a pH below 3 (strongly acidic). Confirmation with a second pH-sensitive dye, bromocresol green allowed mapping a similar acidic pH at the anode. For both dyes, a homogenous, circular simulated soft tissue section of high/low pH evolved around the implant insertion hole.

**Figure 4:**
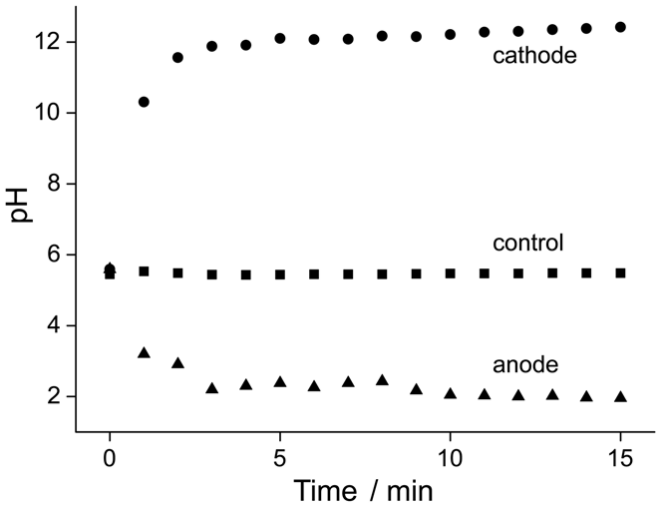
Quantitative pH evolution in the saline around the implant. The evolution of pH in a larger saline environment around the implant measured each minute by a pH microelectrode confirmed the opposing pH drifts at the two implants serving as electrodes.

To illustrate what happens during the electrochemical disinfection in the vicinity of the implant, a number of well-established physico-chemical methods was used. Doping the gelatin with pH-sensitive dyes allowed mapping local changes in acidity (low pH) or alkalinity (high pH), that can both contribute to local implant disinfection. After electrochemical treatment, both pH sensitive dyes changed their color in the appropriate pH region. Thymol blue has two color transitions, from pink over yellow to blue (from low to high pH). Bromocresol green changes from blue to yellow at pH: 4–5. At the cathode, thymol blue indicated an alkaline environment above pH: 9 (Fig. 3). With increasing treatment time, the zone of changed pH increased and formed a circular high-pH area around the implant after 15 minutes (see Fig. 3, top right). On the counter electrode (anode) a low pH (<3) was confirmed locally by the pH-sensitive dye's change to pink. The pronounced local changes in pH were confirmed by using a second pH-sensitive dye. Bromocresol green also indicated an acidic environment at the anode (Fig. 3). A constant circle of yellow-tinted gelatin was observed after 15 minutes of treatment. Quantitative pH measurement with a pH-sensitive electrode revealed a rapid and pronounced drop (pH: ∼2) at the anode as opposed to an increase at the cathode (pH: ∼12) after starting the electrochemical treatment (Fig. 4).

**Figure 5:**
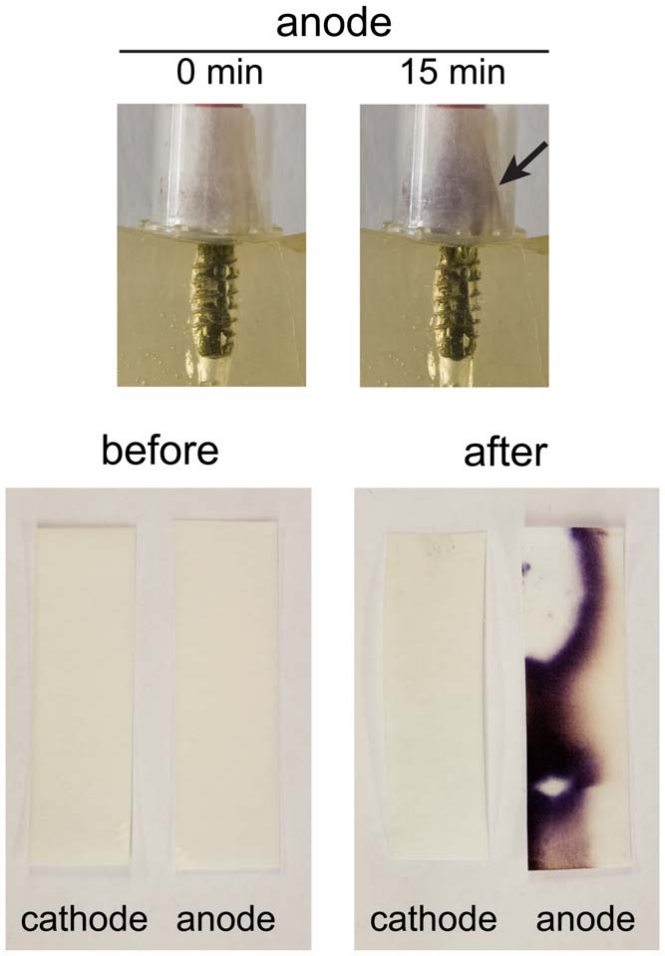
Production of oxidizing species in the vicinity of the implants. Potassium iodide-starch paper before and after the treatment of implants in simulated soft tissue showed a dark blue coloration for the anode after electrolysis. This is in agreement with electrochemical oxidation taking place exclusively at the anode.

The presence of oxidizing species was locally monitored by the use of potassium iodide-starch paper [17]. This well established and sensitive method relies on the oxidation of iodide to molecular iodine (oxidizer +2I^−^ → I_2_+ red. oxidizer) and subsequent formation of I_3_^−^ ions (I^−^+I_2_ → I_3_^−^), that form a highly-colored complex with starch (dark blue areas in Fig. 5). If KI-starch paper was placed above both electrodes, the dark blue color appeared only at the anode (i.e. the site of electrochemical oxidation). The staining was only visible on the inner side of the paper, which was exposed to the oxidative species deriving from the electrolysis (Fig. 5).

## Discussion

Similar to teeth, oral implants are at risk of becoming colonized by biofilms that cause inflammation of their supporting tissues. Failing implants are associated with microbial colonization [18]. In the current study, a common approach for water purification was adapted to kill bacteria adhering to dental implants. In agreement with the hypothesis of this investigation, *in situ* generated active oxidants at the anode (Fig. 1) indeed caused higher bacterial reduction than the mere alkaline environment emerging at the cathode.

Implants that served as cathode showed a maximum reduction of 2 orders of magnitude for the electrochemical treatment (Fig. 2). This reduction can be attributed to the alkaline environment generated at this electrode (Fig. 1), which is only partially tolerated by microorganisms. In addition to mere alkalinity, the production of reactive oxygen species could have equally contributed to bacterial reduction [19]. At the anode, *E. coli* counts were statistically similar to the cathode for 2 mA and 5 mA, but treatment with 7.5 mA and 10 mA resulted in complete implant disinfection (Fig. 2). Similar results have been reported for a process called electro-sterilization that was used for root canal disinfection in the early 20^th^ century [20]. For this treatment the anode was placed in the root canal because the antibacterial effect at the positive electrode (anode) was always distinctly greater than that at the cathode. With reasonable confidence, the here-observed disinfection at the anode can be attributed to the evolution of oxidative species (Fig. 5), such as aquatic chlorine formed by the electrolysis of saline. In addition to the chlorine species, hydroxide radicals or reactive oxygen species could have influenced the inactivation of *E. coli* at the implants serving as anode (Fig. 1). It has been shown that electrical current has a lethal activity on *E. coli* in medium. Besides the electrical current, chlorine can also be essential for the development of a killing effect in a treated medium [21]. Furthermore, not only inactivation of bacteria but also their detachment from the implant during the treatment could contribute to an antimicrobial effect at both electrodes [22]. The amount of electrolysis products is proportional to the amount of electrical charge, which could lead to an enhanced killing efficiency for increasing current, as was observed for the cathode and anode.

Compared to a clinical situation, where a mixture of microbial species is present at the implant site, only a single strain of *E. coli* was used in this study. Facultative enteric bacteria such as enterococci and escherichiae are commonly less susceptible to disinfectants than anaerobic taxa [16], [23], which are seen as the main causative agents of peri-implant disease [15]. However, single-species biofilms of facultative enteric bacteria can be as resistant to disinfectants as mixed-species biofilms [16], [23], and are thus ideal for this type of laboratory investigation. Nevertheless, future studies should also test the electrochemical disinfection of titanium implants contaminated by mixed biofilms, which represent the clinical situation more closely.

Water as well as urinary catheters have been treated with electrical current to reduce bacterial colonization [9], [14]. Both treatments used a continuous current set-up where either higher (water) or lower (catheter) currents were applied. However, for both methods undivided electrodes have been used. In this study, the electrolytic solution around the implants was separated from each other so that two different environments could evolve (Fig. 3). Davis *et al.* [13] reported that a saline solution became more basic during a similar treatment – an observation which is only comparable to one electrode in this study. The possible reason for the two different environments might be the spatial separation in the current investigation. This set-up mimicks the clinical situation, in which the implant (electrode) would also be spatially separated from the counterelectrode. The pH-sensitive dye in the gelatin clearly showed that an alkaline and acidic environment could evolve under these conditions (Fig. 3). At both electrodes the front of changed pH constantly grew with time.

In water treatment, hypochlorite or chlorine are routinely measured with the so-called DPD method [24]. That test, however, is very sensitive to the acidic environment that evolved around the anode implant and hence could not be applied. Normal hypochlorite or aquatic chlorine is stable at basic conditions [25], which were not present in the environment around the anode under the current conditions (Fig. 3 and 4). It has been reported by Czarnetzki *et al.* [26], who analyzed the evolution of hypochlorite, chlorate and oxygen from the electrolysis of a NaCl solution, that it was necessary to compensate the pH of the anolyte with an alkaline solution. This, however, was obviously not indicated in this study because we ultimately intend to directly use electrochemical implant disinfection in a living tissue environment. The *in situ* generated chlorine species have most probably reacted with constituents of the gelatin, or other species [13]. Furthermore, gaseous chlorine could have escaped from the electrolyte. Potassium iodide-starch paper was used to assess oxidant evolution (Fig. 5). The dark blue coloration proves that iodide had been oxidized, which unambiguously confirms oxidant formation. It must be noted that this method can not distinguish between hypochlorite or chlorine evolution. However, since only chlorine is gaseous and the oxidant must have left the liquid to reach the KI-starch paper, it is likely to assume the presence of chlorine (Cl_2_) in this system.

The here applied current was between 2 mA and 10 mA, a range which is tolerable for human beings assuming a covered distance of the electric current in the human body (from the hand to the feet), according to the standard [27]. There is no physiological impact expected for this range of current. In fact, similar currents were routinely applied during electromedication in root canal treatments, using a hand-held cathode and a root canal instrument as the anode [28]. However, aiming for a new possibility to treat peri-implantitis, further experiments have to be carried out due to the difference in distance and tissue between the above mentioned standard and the conditions in this study. The current was set and the resistance was given by the implant, gelatin and saline. The voltage tuned itself in between 4 and 20 V with increasing treatment time, so that the resistance varied between 2 and 6 kΩ. This electrical resistance is similar to the one reported between a root canal terminus and a connected lip clip [29]. In contrast to the reported iontophoresis for urinay catheters with a much lower current (microampere range) [14], the here presented disinfection treatment is much quicker and thus potentially feasible during a dental visit.

Future studies should aim to assess the current approach in an animal model for peri-implantitis.
